# Nonlinear dynamics of *Nosema ceranae* and the fragile resilience of honeybee colonies under environmental strain

**DOI:** 10.1038/s41598-026-45351-1

**Published:** 2026-03-27

**Authors:** Amer M. Salman, Mohd Hafiz Mohd, Ammar Khazaal Kadhim Almansoori, Kawa M. A. Manmi

**Affiliations:** 1 School of Mathematical Sciences, Universiti Sains Malaysia, 11800 USM Gelugor, Penang Malaysia; 2 Centre for Chemical Biology (CCB), Universiti Sains Malaysia, SAINS@USM,29 Block B, 10, Persiaran Bukit Jambul, Bayan Lepas, 11900 Bayan Lepas, Penang Malaysia; 3 Mathematics Institute, University of Warwick, Gibbet Hill Road, Coventry, CV4 7AL UK; 4 Department of Mathematics, College of Science, Salahaddin University-Erbil, Kurdistan Region, Iraq

**Keywords:** *Nosema ceranae* dynamics, Mathematical ecology of pollinators, Bifurcation and stability analysis, Colony collapse and resilience, Eco-epidemiological modelling, Computational biology and bioinformatics, Diseases, Ecology, Ecology, Mathematics and computing, Systems biology

## Abstract

The health and sustainability of honeybee populations are essential for global food production and ecological balance. More than one-third of agricultural crops depend on pollination, making honeybees indispensable to the global economy. However, colonies worldwide are increasingly threatened by the gut parasite *Nosema ceranae*, whose recurrent outbreaks cause severe productivity losses and economic damage to the apicultural and agricultural sectors. Despite its prevalence, the nonlinear mechanisms responsible for the persistence and resurgence of *Nosema ceranae* remain poorly understood. This study aims to develop a mathematical model that interpret the complex epidemiological behaviour of *Nosema ceranae* under realistic biological conditions, specifically focusing on renewal of colony-level susceptibility and the strained resources available for control and management. A nonlinear Susceptible-Infected-Recovered-Susceptible (SIRS) model incorporating renewal of colony-level susceptibility and resource saturation is formulated and analysed using stability theory and numerical bifurcation techniques. Analytical derivations identify the conditions for transcritical and Hopf bifurcations, while numerical continuation analysis and time series simulations validate the theoretical predictions and reveal the rich dynamical structure. The analysis uncovers two critical thresholds governing colony dynamics. A forward (transcritical) bifurcation marks the transition from disease eradication to endemic persistence, while a Hopf bifurcation arising from resource limitations induces sustained oscillations representing recurrent infection waves. The coexistence of stable equilibria and periodic orbits highlights bistability, indicating that small perturbations or change in control interventions can trigger large amplitude outbreaks. These nonlinear feedbacks provide a mechanistic explanation for the cyclical prevalence of *Nosema ceranae* observed in distinct colonies. The proposed framework establishes, for the first time, how the interplay between partial recovery and limited resource availability can drive complex epidemic patterns in honeybee colonies. Beyond its theoretical contribution, the study provides actionable insight for the agriculture industry: efficient allocation of control resources and timely interventions are essential to prevent recurrent epidemics that threaten pollination services and agricultural productivity. The results bridge mathematical modelling and ecological management, offering a predictive foundation for mitigating the economic and environmental impact of *Nosema ceranae* on global food security.

## Introduction

Honeybees are among the most important pollinators sustaining terrestrial ecosystems and global food production, contributing to the pollination of approximately one-third of the crops consumed by humans^1,2^. In recent decades, however, widespread declines in honeybee populations have raised serious ecological and economic concerns^3–6^. These losses are attributed to multiple interacting stressors, including habitat degradation, pesticide exposure, nutritional stress, and pathogenic infections^4,7^. Among these, *Nosema ceranae*, a microsporidian parasite of adult worker bees, has emerged as a dominant pathogen contributing to colony weakening and collapse across temperate and tropical regions^8–10^. Originally a parasite of the Asian honeybee *Apis cerana*^11^, *Nosema ceranae* has successfully crossed the species barrier to infect the western honeybee *Apis mellifera*^12^, establishing a global distribution and replacing its congener *Nosema apis* as the predominant microsporidian infection in managed colonies^13^.

A defining feature of *Nosema ceranae* infection is its chronic and recurrent nature^10,14^. The parasite infects the epithelial cells of the midgut, leading to digestive impairment, reduced nutrient absorption, and premature worker mortality. Spores released through faecal excretion contaminate hive surfaces and food stores, enabling new infections through ingestion by healthy nestmates^12^. This indirect faecal-oral and trophallactic transmission mechanism allows *Nosema ceranae* to persist within colonies even under hygienic management conditions^15^. In general, management interventions and physiological responses can reduce infection intensity or temporarily restore colony function, although environmentally persistent spores frequently remain within the hive structure. Consequently, observed reductions in infection prevalence do not necessarily indicate permanent elimination of the parasite, but rather reflect transient suppression of infection intensity combined with ongoing environmental exposure. Renewed susceptibility may therefore arise through continued spore presence, waning physiological compensation, or the recruitment of newly emerged workers into a contaminated environment. These processes generate effective cycles of decline and resurgence in infection prevalence, supporting long-term persistence of the parasite within colonies^16–18^.

Seasonal fluctuations in colony demography and foraging activity further modulate these infection dynamics, often producing periodic increases in prevalence during spring and summer^19,20^. A further challenge in the management of *Nosema ceranae* arises from the limited capacity for treatment and intervention at both the colony and apiary levels^21^. Control measures such as fumagillin application, nutritional supplementation, and hive sanitation can temporarily reduce infection intensity, yet their effectiveness is constrained by labour, cost, and logistical limitations^22^. In large-scale beekeeping operations, it is rarely feasible to treat all colonies simultaneously or continuously, leading to delayed responses and heterogeneous control outcomes. This limitation can be conceptualised as a form of resource saturation, whereby the effective rate of treatment decreases when the number of infected individuals exceeds the available management capacity^21^. Importantly, such constraints operate at the level of colony and apiary management, reflecting limits in intervention capacity rather than treatment of individual bees. The inclusion of this nonlinear limitation in epidemiological modelling provides a more realistic representation of real-world disease control scenarios in apiculture^22^.

Although infection occurs at the level of individual worker bees, the epidemiological patterns observed in managed apiaries emerge primarily at the colony scale, where demographic turnover, environmental contamination, and management interventions continuously reshape infection prevalence. Worker bees have relatively short lifespans and are continually replaced through recruitment of newly emerged individuals. Consequently, apparent recovery at the colony level does not strictly correspond to immunological clearance in individual bees, but rather reflects a combination of mortality of infected workers, reduction in spore burden, physiological compensation, and demographic replacement. From a dynamical perspective, transitions in colony infection status therefore arise from population turnover and environmental feedbacks as much as from individual-level biological processes^23,24^. Thus, in this study, we adopt a functional colony-level epidemiological perspective in which the model compartments describe proportions of the colony workforce in different infection states. Transitions between these states represent effective changes in colony infection status driven by demographic renewal, treatment-mediated suppression of infection, and persistence of spores within the hive environment, rather than permanent recovery or reinfection of the same individual bee. This modelling framework is consistent with the ecological and management processes that govern disease persistence in honeybee colonies.

While several epidemiological models have been developed to study *Nosema ceranae* infections or general bee pathogens, most frameworks remain limited to either individual-level infection processes or simplified colony-level dynamics with constant recovery rates and unlimited treatment capacity^25,26^. Such assumptions overlook the complex feedbacks between infection intensity, resource allocation, and management constraints that characterise real apiary conditions. The nonlinear SIRS formulation introduced in this study addresses this gap by explicitly integrating partial infection suppression and resource saturation into a unified colony-level framework^27,28^. This approach captures how limited intervention capacity and transient reductions in infection burden can give rise to oscillatory infection patterns and bistable outcomes that are frequently observed in managed honeybee populations but not reproduced by classical models^29–31^.

The contribution of this research lies in the development and rigorous analysis of a nonlinear SIRS-type model tailored to represent colony-level dynamics of *Nosema ceranae* under the combined influence of recurrent infection pressure and constrained management resources. The proposed framework links intervention limitations directly to epidemiological dynamics through a nonlinear treatment response, enabling systematic investigation of how resource availability and infection feedbacks jointly shape colony outcomes. From a dynamical systems perspective, the analysis reveals a range of qualitative transitions, including forward (transcritical) bifurcations, Hopf bifurcations, and regions of bistability, providing a mechanistic interpretation of recurrent infection cycles and stability shifts in managed colonies. By integrating biologically grounded colony-level processes with nonlinear stability analysis, the model offers a coherent theoretical framework for understanding how demographic turnover, environmental persistence of pathogens, and finite management capacity interact to determine long-term infection behaviour. These insights contribute to a deeper conceptual understanding of colony resilience, instability thresholds, and the dynamical conditions under which recurrent outbreaks may emerge or be suppressed.

The remainder of this paper is organised as follows. Section “The model formulation” presents the formulation of the *Nosema ceranae* SIRS model and its biological interpretation. Section “Characterisation of equilibrium points” provides a detailed mathematical analysis of the stability, equilibrium points, and bifurcation structure. Section “Results and discussion” reports the numerical simulations, validating the analytical results and illustrating the impact of control measures. Finally, section “Conclusion and implications” discusses the biological implications of the findings and highlights potential directions for future research on sustainable *Nosema ceranae* control in honeybee colonies.

## The model formulation

To investigate the combined influences of limited treatment capacity, management constraints, and transient infection suppression on the transmission dynamics of *Nosema ceranae* in honeybee colonies, we consider a generalised form of the classical Susceptible-Infected-Recovered (SIR) framework together with renewal of colony-level susceptibility processes^27^. *Nosema ceranae* is a microsporidian parasite that infects the midgut epithelial cells of adult worker bees, leading to impaired digestion, reduced foraging efficiency, shortened lifespan, and progressive colony weakening. The parasite spreads predominantly through the faecal-oral route and trophallactic contact, as infected bees shed environmentally resistant spores that are subsequently ingested by healthy nestmates^14,32^. Within a densely populated colony, such indirect horizontal transmission enables the pathogen to persist even under hygienic management and seasonal variations in bee activity.

The present model adopts a functional colony-level epidemiological perspective. Rather than representing immunological transitions of individual bees, the model describes the evolving distribution of the colony workforce across different infection states. Transitions between these states reflect demographic turnover, environmental persistence of spores, and management-mediated suppression of infection intensity. This formulation captures effective changes in colony infection status that arise from population renewal, physiological compensation, and intervention effects, which together govern observed infection prevalence at the colony scale.

**Fig. 1:**
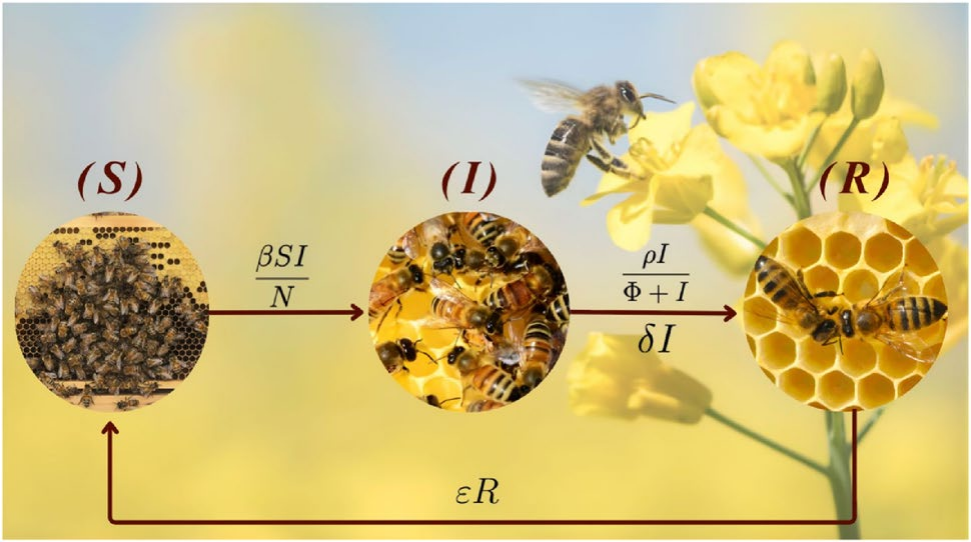
Diagrammatical representation of the SIRS model for the *Nosema ceranae* transmission dynamics.

To capture these biological processes and their interaction with limited treatment interventions at the colony or apiary level, the model is illustrated diagrammatically in Fig. 1. The present framework adopts a system of ordinary differential equations of the SIRS type^28,29^:

1 $$\begin{aligned} {\left\{ \begin{array}{ll} \dfrac{dS}{dt} = \eta N - \dfrac{\beta S I}{N} + \varepsilon R - vS,\\[6pt] \dfrac{dI}{dt} = \dfrac{\beta S I}{N} - (\delta + v)I - \dfrac{\rho I}{\Phi + I},\\[8pt] \dfrac{dR}{dt} = \delta I + \dfrac{\rho I}{\Phi + I} - (\varepsilon + v)R,\\[6pt] N = S + I + R, \end{array}\right. } \end{aligned}$$

where *N* denotes the total adult worker population of the colony, subdivided into three functional epidemiological compartments.

In the present formulation, the total colony population *N* is assumed to remain constant over the time horizon of the analysis. This assumption is intended as a simplifying approximation that allows the dynamical effects of infection, recovery, and limited management capacity to be isolated without the additional complexity of demographic feedback^33,34^. Biologically, this reflects a quasi-stationary colony phase in which recruitment and mortality are approximately balanced over the infection timescales considered. Under such conditions, the model represents the redistribution of individuals among functional health states within a stable workforce rather than large-scale changes in colony size. This approximation is also consistent with the well-documented social buffering mechanisms exhibited by eusocial insects, whereby behavioural and organisational adjustments can temporarily stabilise the effective workforce despite environmental fluctuations^35,36^. The susceptible class, *S*(*t*), represents the fraction of the colony workforce that is functionally healthy yet vulnerable to infection through environmental exposure to spores, trophallactic contact, or ingestion of contaminated food resources. The infected class, *I*(*t*), represents the fraction of the workforce actively harbouring *Nosema ceranae* and contributing to environmental spore accumulation within the hive. The recovered class, *R*(*t*), represents the fraction of the workforce that is functionally restored following infection suppression or reduction in parasite burden. This state may arise through treatment effects, physiological compensation, or demographic replacement of infected individuals by newly emerged workers. Importantly, this classification reflects temporary restoration of colony function rather than permanent immunological recovery of individual bees. Transitions from *R* back to *S* represent renewal of colony-level susceptibility driven by persistent environmental contamination, waning physiological compensation, or recruitment of new workers into a spore-containing environment, rather than reinfection of the same individual bee.

In system (1), the parameter $\eta$ denotes the per capita recruitment rate of newly emerged adult workers, while *v* represents the natural mortality rate. These processes collectively regulate population turnover within the colony. The model assumes that recruitment and mortality balance over the time scale of infection dynamics, yielding a quasi-stationary total population size. This approximation allows the analysis to focus on infection redistribution within the workforce rather than long-term colony growth. The transmission parameter $\beta$ quantifies the effective rate at which susceptible workers acquire infection through exposure to environmentally deposited spores and trophallactic interactions. This parameter therefore incorporates both contact intensity and contamination level within the hive environment. The parameter $\delta$ represents the effective rate at which infection intensity is reduced sufficiently for individuals to be reclassified into the functionally restored workforce. This transition may occur through treatment-mediated reduction in parasite load, physiological recovery that restores functional capacity, or replacement of infected workers through demographic turnover. The parameter $\varepsilon$ represents the rate at which temporary functional restoration is lost, returning individuals to a susceptible state. This transition reflects renewal of colony vulnerability due to environmental persistence of spores, waning physiological compensation, or recruitment of new workers into a contaminated environment.

The nonlinear term

$$\begin{aligned} \dfrac{\rho I}{\Phi + I} \end{aligned}$$

models the effect of limited treatment or management capacity, originally introduced by Zhou and Fan^37^. Here, $\rho$ denotes the maximum achievable intervention intensity, while $\Phi$ is the half-saturation constant describing how rapidly treatment effectiveness declines as infection burden increases. This nonlinear response represents constraints in colony- or apiary-level management, including limits in labour, treatment availability, monitoring capacity, and intervention timing. As infection burden rises, available management resources are distributed across a larger number of affected individuals or colonies, reducing the effective per-capita impact of treatment. The saturation mechanism therefore describes diminishing intervention efficiency at the management scale rather than treatment limitation at the level of individual bees.

In the absence of renewal of colony-level susceptibility and treatment constraints (i.e. $\varepsilon = \rho = 0$), the system reduces to the classical SIR model. All parameters are assumed non-negative to ensure biological feasibility. The model formulated in Equation (1) extends the classical SIR framework by incorporating two ecologically grounded mechanisms that are central to colony-level *Nosema ceranae* epidemiology: transient functional restoration of the workforce and nonlinear limitations in intervention capacity. These processes arise naturally from demographic turnover, environmental persistence of spores, and finite management resources. By embedding these mechanisms within a unified dynamical framework, the model captures how feedbacks between infection burden and intervention capacity can generate persistent infection, oscillatory outbreaks, and alternative stable regimes. Such behaviours reflect the complex interaction between pathogen transmission, colony renewal, and management response that characterises real-world apicultural systems.

## Characterisation of equilibrium points

In this section, we will investigate the stability analysis of the SIRS model (1). Since the population of *R* can be estimated based on *N*, *S* and *I*,(i.e. $R=N-(S+I)$). So, the model (1) will be reduced to the following system of ordinary differential equations:

2 $$\begin{aligned} {\left\{ \begin{array}{ll} \frac{dS}{dt} =\eta N -\frac{\beta S I}{N} +\varepsilon (N-(S+I)) -vS\\ \frac{dI}{dt} =\frac{\beta S I}{N} - (\delta + v)I - \frac{\rho I}{\Phi + I} \\ \end{array}\right. } \end{aligned}$$

### Existence of the equilibrium points

To calculate the equilibrium points of model (2), we denote each of the equations in the dynamical system (2) as follows:

3 $$\begin{aligned} F(S, I) = \eta N -\frac{\beta S I}{N} +\varepsilon (N-(S+I)) -vS. \end{aligned}$$

4 $$\begin{aligned} G(S, I) = \frac{\beta S I}{N} - (\delta + v)I - \frac{\rho I}{\Phi + I}. \end{aligned}$$

Now, we set each of the two equations (3) and (4) to be equal to zero, then we can solve for *S* and *I* and obtain distinct equilibria of the system. However, the equilibria of interest are:

(i) The disease-free equilibrium (DFE), $E_0(\frac{N(\eta +\varepsilon )}{v+\varepsilon }, 0)$, where the disease is eliminated as the population of $I^*=0$.
(ii) The endemic equilibrium (EE), $E^*(S^*, I^*)$, where the disease spread in the community and both populations of $S^* > 0$ and $I^* > 0$.

Next, we analyse the stability of equation (2) in details.

### Stability analysis

This section focusses on examining the stability of equilibrium points. Stability analysis for the two equilibrium points were carried out using the Jacobian matrix. To formulate the Jacobian matrix, we take the partial derivative of the equations (3) and (4) as follows.

$$\begin{aligned} {\mathcal {J}}(S, I) = \begin{pmatrix} \frac{\partial F}{\partial S} & & \frac{\partial F}{ \partial I}\\ \frac{\partial G}{\partial S} & & \frac{\partial G}{ \partial I} \end{pmatrix}. \end{aligned}$$

Which yields to,

5 $$\begin{aligned} {\mathcal {J}}(S, I) = \begin{pmatrix} -\frac{\beta I}{N} - (v + \varepsilon ) & & -\frac{\beta S}{N} - \varepsilon \\ \\ \frac{\beta I}{N} & & \frac{\beta S}{N} - \frac{\Phi \rho }{(I+\Phi )^2} - (v+\delta ) \end{pmatrix} \end{aligned}$$

Mathematica software has been utilised to conduct the computation of the Jacobian and equilibrium points. Furthermore, to obtain the characteristics equation and get the eigenvalues of the equilibrium points, we substitute equilibrium points into the Jacobian matrix (5).

#### Stability analysis of the DFE

In this section, we provide the dynamical system analysis associated with DFE point $E_0=(\frac{N(\eta +\varepsilon )}{v+\varepsilon }, 0)$.

Firstly, we shall substitute the DFE point $E_0$ into the Jacobian matrix (5), to obtain,

6 $$\begin{aligned} {\mathcal {J}}(E^0) = \begin{pmatrix} -(v + \varepsilon ) & & -\frac{\beta (\eta +\varepsilon )}{v+\varepsilon } - \varepsilon \\ \\ 0 & & \frac{\beta (\eta +\varepsilon )}{v+\varepsilon } - \frac{\rho }{\Phi } - (v+\delta ) \end{pmatrix}. \end{aligned}$$

Then, we compute the eigenvalues of the matrix (6) which are given by:

(i) $$\lambda _1 = -(v + \varepsilon )$$.
(ii) $$\lambda _2 = -(\frac{\rho }{\Phi } + v+\delta ) + \frac{\beta (\eta +\varepsilon )}{v+\varepsilon }$$.

Recall that we assumed all the parameters are real and positive. So, $\lambda _1 \le 0$. Therefore, the DFE is is asymptotically stable if the quantity $(\frac{\rho }{\Phi } + v+\delta ) > \frac{\beta (\eta +\varepsilon )}{v+\varepsilon }$. Conversely, it is unstable if the quantity $(\frac{\rho }{\Phi } + v+\delta ) \le \frac{\beta (\eta +\varepsilon )}{v+\varepsilon }$.

#### Stability analysis of the EE

In this section, we will study the dynamical behaviour of model (2) associated with the EE. The EE of model (2) is given by:

$$\begin{aligned} E^* = (S^*, I^*), \end{aligned}$$

Then, the Jacobian matrix of the EE point is given by:

7 $$\begin{aligned} {\mathcal {J}}(E^*) = \begin{pmatrix} -\frac{\beta I^*}{N} - (v + \varepsilon ) & & -\frac{\beta S^*}{N} - \varepsilon \\ \\ \frac{\beta I^*}{N} & & \frac{\beta S^*}{N} - \frac{\Phi \rho }{(I^*+\Phi )^2} - (v+\delta ) \end{pmatrix} \end{aligned}$$

##### Lemma 1

*Let* $M = -\text {Tr}({\mathcal {J}}(E^*))$* and* $K = \text {Det}({\mathcal {J}}(E^*))$,* and let*

8 $$\begin{aligned} Q(\lambda ) = \lambda ^2 + M \lambda + K \end{aligned}$$

*be the characteristic equation of eigenvalues associated to the Jacobian matrix* (7) *computed at the EE* ($E^*$). *According to Theorem (Routh-Hurwitz criteria) then the following are hold:*

1. Equation (8) has real negative roots if and only if the following conditions are satisfied:
  - $$M > 0$$.
  - $$M^2 > 4K$$.
  - $$\sqrt{M^2 - 4K} < M$$.
2. Equation (8) has complex roots if and only if the following conditions are satisfied:
  - $$M > 0$$.
  - $$M^2 < 4K$$.

### Bifurcation analysis

In this section, we shall we establish the existence of the forward and Hopf bifurcations for the dynamical system (2).

#### Forward bifurcation

A forward bifurcation, also known as a transcritical bifurcation, occurs in a dynamical system when two equilibrium points exchange their stability as a parameter crosses a critical value. In this scenario, no new equilibria are created or destroyed; instead, the stability of the equilibria changes at the bifurcation point, leading to a smooth transition in the dynamics of the system. In epidemiological modelling basic reproduction number (BRN) is one of the threshold components that often being associated with forward bifurcation. The BRN is denoted as ${\mathcal {R}}_0$ which, represents the average number of secondary infections caused by a single infected individual in an otherwise susceptible population. The term ${\mathcal {R}}_0$ is a critical bifurcation parameter: if ${\mathcal {R}}_0 < 1$ the disease cannot sustain itself and eventually dies out, while if ${\mathcal {R}}_0 > 1$ the disease can invade and establish itself within the population. The transition from a disease-free equilibrium (DFE) to an endemic equilibrium (EE) as ${\mathcal {R}}_0$ increases past 1 is often characterised by a transcritical bifurcation^30^.

##### Theorem 1

*Considering the equilibria* $E_0 =(\frac{N(\eta + \varepsilon )}{v+ \varepsilon }, 0)$ *and* $E^* = (S^*, I^*)$ *computed in section* “Existence of the equilibrium points” *and the basic reproduction number (BRN) of system* (2) is given by:

9 $$\begin{aligned} {\mathcal {R}}_0 = \frac{\beta (\eta + \varepsilon )}{(v+\varepsilon )(\frac{\rho }{\Phi } + v+ \delta )}. \end{aligned}$$

*Then, the following properties hold*:

(i) If ${\mathcal {R}}_0 < 1$, the DFE ($E_0$) is asymptotically stable and the infection dies out over time.
(ii) If ${\mathcal {R}}_0 > 1$, there exist a unique EE ($E^*$) with $I^* > 0$.

##### *Proof*

The stability of the DFE depends on the dominant eigenvalue of the Jacobian matrix at $E_0$. From section “Stability analysis of the DFE” the dominant eigenvalue is given by:

10 $$\begin{aligned} \lambda = -(\frac{\rho }{\Phi } + v + \delta ) + \frac{\beta (\eta + \varepsilon )}{v + \varepsilon }. \end{aligned}$$

Changes in dynamics occur when the dominant eigenvalue crosses zero:

$$\begin{aligned} \lambda = -(\frac{\rho }{\Phi } + v + \delta ) + \frac{\beta (\eta + \varepsilon )}{v + \varepsilon } =0. \end{aligned}$$

The dominant eigenvalue is stable when $\lambda < 0$, thus:

$$\begin{aligned} & -(\frac{\rho }{\Phi } + v + \delta ) + \frac{\beta (\eta + \varepsilon )}{v + \varepsilon }< 0, \\ & \implies \frac{\beta (\eta + \varepsilon )}{v + \varepsilon }< (\frac{\rho }{\Phi } + v + \delta ), \\ & \implies \frac{\beta (\eta + \varepsilon )}{(v + \varepsilon ) (\frac{\rho }{\Phi } + v + \delta )} < 1. \end{aligned}$$

Suppose that $${\mathcal {R}}_0 < 1$$

$$\begin{aligned} & \implies \frac{\beta (\eta + \varepsilon )}{(v+\varepsilon )(\frac{\rho }{\Phi } + v+ \delta )}< 1 \\ & \implies \beta (\eta + \varepsilon ) < (v+\varepsilon )(\frac{\rho }{\Phi } + v+ \delta ) \end{aligned}$$

Since all the parameters are assumed to be positive and the quantity $\beta (\eta + \varepsilon ) < (v+\varepsilon )(\frac{\rho }{\Phi } + v+ \delta )$. Hence, the dominant eigenvalue (10) is always negative when ${\mathcal {R}}_0 < 1$. Conversely, when ${\mathcal {R}}_0 > 1$ becomes unstable, and a stable endemic equilibrium $E^*$ emerges, satisfying $I^* > 0$. $\square$

#### Hopf bifurcation

Hopf bifurcation occurs when a system undergoes a qualitative change in the equilibrium point as a parameter is varied, leading to the creation or disappearance of periodic orbits. Specifically, a supercritical Hopf bifurcation is associated with the transition from a stable fixed point (equilibrium) to an unstable ones, with corresponding creation of a stable periodic orbit. Conversely, a subcritical Hopf bifurcation is associated with the transition from unstable equilibrium point to a stable equilibrium point with the creation of unstable limit cycles. Hopf bifurcation can occur only at (EE).

##### Theorem 2

*System* (2) *undergoes a Hopf bifurcation as the parameter* $\Phi$ *surpasses the transition threshold* $\Phi _{H}$.

##### *Proof*

Recall the characteristic equation (8) of the linearised Jacobian matrix (7), the Hopf bifurcation occurs if the following conditions are hold:

(i) $$[Tr({\mathcal {J}}(E_*))]|_{\Phi =\Phi _{H}} = 0$$
(ii) $$[Det({\mathcal {J}}(E_*))]|_{\Phi =\Phi _{H}} > 0$$
(iii) $$\frac{d}{d\Phi }[Tr({\mathcal {J}}(E_*))]|_{\Phi =\Phi _{H}} \ne 0$$

Firstly, we compute the sum of the diagonal elements i.e., the trace of ${\mathcal {J}}(E^*)$ as follows:

11 $$\begin{aligned} Tr({\mathcal {J}}(E^*)) = -(2v+\varepsilon +\delta ) + \frac{\beta (S^*-I^*)}{N} - \frac{\Phi \rho }{(I^* + \Phi )^2}. \end{aligned}$$

we compute the determinant ${\mathcal {J}}(E^*)$:

12 $$\begin{aligned} |{\mathcal {J}}(E^*)| = -(\frac{\beta I^*}{N} + (v + \varepsilon ))(\frac{\beta S^*}{N} - \frac{\Phi \rho }{(I^*+\Phi )^2} - (v+\delta )) + (\frac{\beta ^2 S^* I^*}{N^2} - \frac{ \varepsilon \beta I^*}{N}) \end{aligned}$$

To prove condition (i), we need to set equation (11) equals to zero and solve for $\Phi = \Phi _{H}$.

$$\begin{aligned} -(2v+\varepsilon +\delta ) + \frac{\beta (S^*-I^*)}{N} - \frac{\Phi _H \rho }{(I^* + \Phi _H)^2} = 0 \end{aligned}$$

First, move all terms not involving $\Phi _H$ to the right-hand side:

$$\begin{aligned} \frac{\Phi _H \rho }{(I^* + \Phi _H)^2} = \frac{\beta (S^*-I^*)}{N} - (2v + \varepsilon + \delta ). \end{aligned}$$

Simplify the right-hand side. Let us define a constant $C$ as:

$$\begin{aligned} C&= \frac{\beta (S^* - I^*)}{N} - (2v + \varepsilon + \delta ), \\ \implies&\frac{\Phi _H \rho }{(I^* + \Phi _H)^2} = C, \\ \implies&\Phi _H \rho = C (I^* + \Phi _H)^2, \\ \implies&\Phi _H \rho = C (I^{*2} + 2I^* \Phi _H + \Phi _H^2), \\ \implies&C \Phi _H^2 + (2 I^* C) \Phi _H + C I^{*2} - \Phi _H \rho = 0, \\ \implies&C \Phi _H^2 + (2 I^* C - \rho ) \Phi _H + C I^{*2} = 0. \end{aligned}$$

Now, we now apply the quadratic formula:

$$\begin{aligned} \Phi _{H} = \frac{-(2 I^* C - \rho ) \pm \sqrt{(2 I^* C - \rho )^2 - 4C \cdot C I^{*2}}}{2C}. \end{aligned}$$

Thus, at $\Phi _H$ is:

$$\begin{aligned} \Phi _{H} = \frac{-(2 I^* C - \rho ) \pm \sqrt{\rho ^2 - 4 I^* C \rho }}{2C} \end{aligned}$$

It can be checked that at $[Tr({\mathcal {J}}(E_*))]|_{\Phi =\Phi _{H}} = 0$ as stated in condition (i).

For condition (ii), we compute the determinant

13 $$\begin{aligned} \implies -(\frac{\beta I^*}{N} + (v + \varepsilon ))(\frac{\beta S^*}{N} - \frac{\Phi \rho }{(I^*+\Phi )^2} - (v+\delta )) + (\frac{\beta ^2 S^* I^*}{N^2} - \frac{ \varepsilon \beta I^*}{N}) \end{aligned}$$

Substitute $\Phi _H$ in (13), then it can be shown that the inequality below holds:

14 $$\begin{aligned} \frac{\beta ^2 S^* I^*}{N^2} > (\frac{\beta I^*}{N} + (v + \varepsilon ))(\frac{\beta S^*}{N} - \frac{\Phi _H \rho }{(I^*+\Phi _H)^2} - (v+\delta )) + (\frac{ \varepsilon \beta I^*}{N}) \end{aligned}$$

By ensuring (14) holds, then we have $[Det({\mathcal {J}}(E_*))]|_{\Phi =\Phi _{H}} > 0$ as stated in condition (ii).

To show condition (iii), we shall take the partial derivative of (i) with respect to the bifurcation parameter $\Phi _H$:

15 $$\begin{aligned} \frac{d}{d\Phi } |Tr(J(E_*))| = \frac{d}{d\Phi } (-2v-\varepsilon +\delta + \frac{\beta (S^*-I^*)}{N} + \frac{\Phi \rho }{(I^* + \Phi _H)^2}). \end{aligned}$$

Then substitute $\Phi = \Phi _{H}$ into (15)

16 $$\begin{aligned} \frac{d}{d\Phi _H} |Tr(J(E_*))| = \frac{d}{d\Phi _H} (-2v-\varepsilon +\delta + \frac{\beta (S^*-I^*)}{N} + \frac{\Phi _H \rho }{(I^* + \Phi _H)^2}). \end{aligned}$$

Solving the equation above (16),

17 $$\begin{aligned} \frac{d}{d\Phi _H} |Tr(J(E_*))| = \frac{\rho (I^* - \Phi _H)}{(I^* + \Phi _H)^3}, \end{aligned}$$

where we can conclude that $|\frac{d}{d\Phi _H} (Tr(J(E_*)))|_{\Phi _H} \ne 0$. Therefore, the Hopf bifurcation occurs around the EE at $\Phi = \Phi _H$. $\square$

## Results and discussion

In this section, we present the results of the numerical bifurcation analysis and the corresponding time series simulations for the SIRS model (1) governing the transmission of *Nosema ceranae* in honeybee colonies. The bifurcation results were obtained using the XPPAUT continuation package, while the numerical simulations were performed using MATLAB under the parameterisation given in Table 1. The focus of the analysis is on the two key parameters $\rho$ and $\Phi$, which represent the maximum treatment capacity and the half-saturation constant describing the efficiency of resource utilisation. These parameters encapsulate the constraints of limited management and treatment resources in apiary systems. Through a combined analysis of one-parameter and two-parameter continuations, we identify the critical thresholds at which equilibrium states lose stability, leading to the emergence of sustained oscillations in the infected population. We then demonstrate that the numerical simulations are in excellent qualitative agreement with the bifurcation predictions, thereby validating the analytical results derived in section “Characterisation of equilibrium points”. The baseline parameter values used in the simulations are selected from biologically plausible ranges reported in the literature^38^ and are intended to represent a typical managed colony under moderate infection pressure. Recruitment and mortality rates ($\eta$ and *v*) are chosen to reflect approximate daily workforce turnover in stable colonies, while the transmission rate $\beta$ and recovery rate $\delta$ are consistent with experimentally observed infection progression and partial suppression under treatment. The reinfection parameter $\varepsilon$ is set at a smaller magnitude relative to mortality to represent gradual renewal of colony-level susceptibility driven by demographic turnover and environmental persistence of spores rather than rapid individual reinfection. The management-related parameters $\rho$ and $\Phi$ are explored over wide intervals to capture both efficient and resource-limited intervention regimes. Importantly, the qualitative dynamical behaviours reported in this study are robust across neighbouring parameter values, as supported by the sensitivity analysis in section “Sensitivity of dynamical behaviour to model parameters”.

**Table 1:** Model parameters, descriptions, and baseline values used for simulating the transmission dynamics of *Nosema ceranae* in a honeybee colony.

Parameter	Symbol	Value	Unit
Workforce recruitment rate (emergence of new adult workers)	$\eta$	0.03	$\hbox {day}^{-1}$
Natural mortality rate	*v*	0.03	$\hbox {day}^{-1}$
Effective colony transmission intensity (within-hive spore exposure)	$\beta$	2.10	$\hbox {day}^{-1}$
Infection suppression rate (treatment, removal and functional restoration)	$\delta$	0.10	$\hbox {day}^{-1}$
Renewal of colony-level susceptibility (environmental re-exposure and demographic turnover)	$\varepsilon$	0.006	$\hbox {day}^{-1}$
Maximum achievable intervention intensity	$\rho$	Vary	
Half-saturation constant	$\Phi$	Vary	
Total effective workforce size	*N*	800	

The parameter values setting is based on study^38^.

### Numerical continuation results

**Fig. 2:**
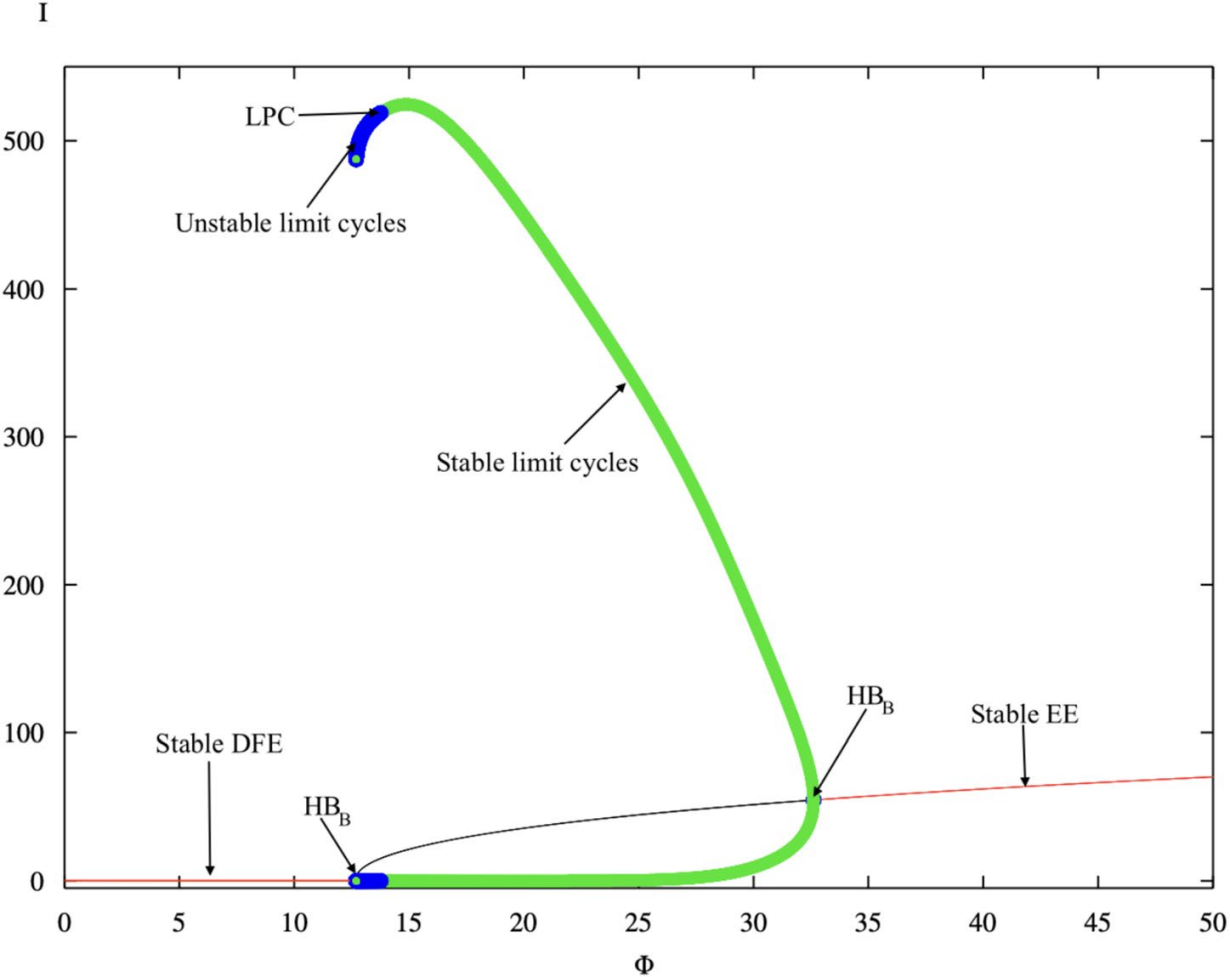
One-parameter continuation of the infected equilibrium with respect to $\Phi$. The diagram exhibits subcritical and supercritical Hopf bifurcations depending on the resources efficiency. Green curves represents stable periodic orbits, while blue curve represents the unstable stable periodic orbits. red and black represent stable and unstable equilibria respectively.

The one-parameter continuation of the infected equilibrium with respect to the treatment efficiency parameter $\Phi$ is presented in Fig. 2. The parameter $\Phi$ quantifies the degree to which available treatment and hygienic management efforts are effectively distributed within the colony. Smaller values of $\Phi$ correspond to higher efficiency, where control measures such as hygienic behaviour, comb replacement, or supplementary feeding are applied consistently and with minimal delay, allowing the infection to be mitigated effectively.

As $\Phi$ decreases, the infected equilibrium $I^{*}$ loses stability at a critical value $\Phi _{H}$, where a Hopf bifurcation arises and gives birth to a branch of stable periodic oscillations (green curve). This transition indicates the onset of oscillatory infection dynamics, corresponding to recurrent *Nosema ceranae* outbreaks within the honeybee colony. For $\Phi > \Phi _{H}$, the endemic equilibrium remains locally stable, representing a steady state infection level in which the parasite persists chronically at a constant prevalence. In contrast, when $\Phi < \Phi _{H}$, inefficient treatment or irregular management destabilises the equilibrium, producing periodic infection waves driven by fluctuating spore loads and renewal of colony-level susceptibility events. The smooth emergence of stable limit cycles from the equilibrium indicates a supercritical Hopf bifurcation, implying that the transition from stability to oscillation occurs gradually. Biologically, this phenomenon reflects the field observations that *Nosema ceranae* infections tend to oscillate seasonally or cyclically when management actions such as fumagillin application, feeding supplementation, or hive sanitation are inconsistently implemented. The Hopf threshold $\Phi _{H}$ therefore delineates a critical boundary between stable endemic infection and recurrent epidemic dynamics within managed colonies.

**Fig. 3:**
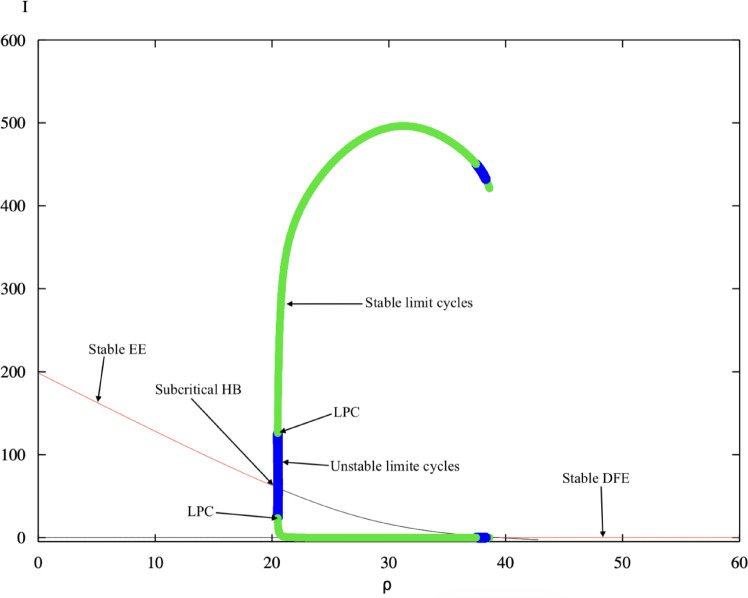
One-parameter continuation of the infected equilibrium with respect to $\rho$. The diagram exhibits subcritical and supercritical Hopf bifurcations depending on the resource supply rate. Green curves represent stable periodic orbits, blue curves represent unstable periodic orbits, while red and black denote stable and unstable equilibria respectively.

The continuation of equilibria with respect to the treatment capacity $\rho$ is depicted in Fig. 3. The parameter $\rho$ represents the overall capacity of the colony or the beekeeper to apply management interventions, encompassing hygienic behaviour, spore-cleansing activity, and the ability of the beekeeper to distribute treatments effectively across hives.

For small values of $\rho$, corresponding to limited treatment capacity, the system undergoes a subcritical Hopf bifurcation. In this regime, the infected equilibrium does not simply lose stability; instead, it coexists with both stable and unstable periodic solutions, generating a well-defined region of bistability bounded by a Limit Point of Cycles (LPC). Within this parameter window, two stable attractors are present simultaneously: a stable endemic equilibrium and a stable large-amplitude limit cycle. The phase-space geometry is organised by an unstable limit cycle that forms the basin boundary separating these two long-term outcomes. Trajectories initiated inside this unstable periodic orbit converge towards the endemic equilibrium, whereas those outside its boundary are repelled towards the oscillatory attractor. Hence, for identical parameter values, the colony’s long-term fate depends sensitively on its initial infection abundance. Numerical continuation further clarifies that this unstable periodic orbit originates from the subcritical Hopf bifurcation and persists until it collides with the stable limit cycle at the LPC. This collision marks the disappearance of sustained oscillations and delimits the parameter interval in which bistability occurs. Beyond this threshold, the endemic equilibrium becomes the unique attractor of the system. The loss of oscillatory behaviour therefore occurs through a global reorganisation of invariant sets rather than through gradual amplitude reduction.

Biologically, this bistable structure implies that colony resilience is governed not only by management parameters but also by perturbation magnitude. When the colony operates within this bistable region, small infectious disturbances may decay towards a chronic yet stable endemic state. However, perturbations exceeding the unstable separatrix may drive the system towards recurrent large-amplitude infection cycles characterised by alternating periods of decline and resurgence in *Nosema* intensity. Colonies situated near this dynamical threshold may therefore appear stable under routine conditions, yet remain vulnerable to sufficiently strong epidemiological or environmental shocks that push the system across the basin boundary.

As $\rho$ increases, the subcritical Hopf transition gradually transforms into a supercritical one, indicating that enhanced treatment availability and more frequent management interventions suppress bistability and reduce oscillatory behaviour. When $\rho$ exceeds a critical value $\rho _{H}$, the endemic equilibrium becomes globally stable and all oscillations vanish. This transition demonstrates that adequate treatment capacity stabilises the colony, leading to a steady endemic equilibrium where infection is persistently low. From an epidemiological and apicultural standpoint, this bifurcation behaviour indicates that insufficient management resources are a major driver of cyclic *Nosema ceranae* outbreaks. Enhancing treatment capacity, either through improved colony hygiene or timely interventions, is therefore essential to stabilising disease dynamics and maintaining colony health.

The analytical Hopf bifurcation threshold derived in Theorem 3.3 provides a precise theoretical prediction for the onset of oscillatory dynamics in the colony infection system. In particular, the critical value $\Phi _H$ obtained from the Hopf condition marks the parameter level at which the endemic equilibrium loses stability through a pair of complex conjugate eigenvalues crossing the imaginary axis. This analytical prediction is directly corroborated by the numerical continuation results shown in Figs. 2 and 3. In both diagrams, the transition from a stable endemic equilibrium to sustained oscillatory behaviour occurs precisely at the parameter value identified by the Hopf threshold. The bifurcation point detected numerically as a Hopf bifurcation in the XPPAUT continuation curves therefore coincides with the analytically derived stability boundary, confirming the internal consistency between the theoretical analysis and the numerical bifurcation structure.

**Fig. 4:**
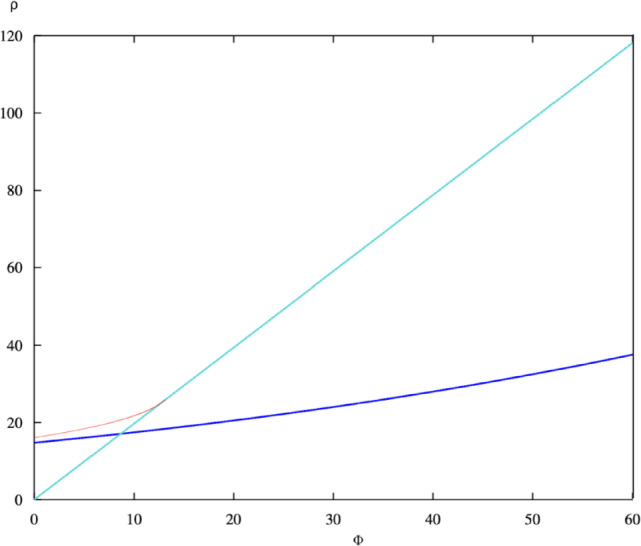
Two-parameter bifurcation diagram in the $(\Phi , \rho )$ plane showing Hopf loci. The curve separates stable equilibrium (below) from oscillatory regimes (above). The region of bistability is indicated near the transition boundary.

To explore the combined influence of treatment efficiency $\Phi$ and treatment capacity $\rho$, a two-parameter bifurcation analysis was conducted, and the results are shown in Fig. 4. The Hopf bifurcation curve divides the $(\Phi , \rho )$ plane into regions corresponding to qualitatively distinct dynamical behaviours. Below the Hopf locus, the endemic equilibrium is asymptotically stable, representing colonies in which infection persists at a controlled and predictable level. Above the curve, the system exhibits stable periodic oscillations, indicating recurrent infection and respectively recovery cycles that can destabilise the colony. A narrow region near the bifurcation boundary indicates bistability, where both a stable equilibrium and a stable limit cycle coexist. The orientation of the Hopf curve reveals a clear trade-off between treatment efficiency and capacity: as efficiency ($\Phi$) decreases, greater treatment capacity ($\rho$) is required to sustain stability. Conversely, when treatment capacity diminishes, stability can be preserved only through improved efficiency of management. This interplay demonstrates that colony health depends jointly on both the availability of resources and the precision with which they are deployed.

From a biological perspective, this two-parameter diagram encapsulates the critical management regimes for controlling *Nosema ceranae*. Colonies operating below the Hopf boundary represent resilient systems capable of maintaining stable endemic infection levels, whereas those above the boundary are vulnerable to cyclic outbreaks and possible collapse. The $(\Phi , \rho )$ plane thus serves as a predictive tool for identifying safe operational zones for colony management. By optimising both treatment efficiency and capacity, beekeepers can effectively mitigate oscillatory outbreaks and ensure long-term colony stability.

### Dynamical behaviour and attractor structure of the model

To establish the qualitative behaviour of the proposed SIRS framework, numerical simulations were conducted using representative parameter sets drawn from distinct regions of parameter space identified through bifurcation analysis. The simulations reveal that the model supports three fundamentally different long-term epidemiological outcomes: infection elimination, persistent endemic infection, and sustained recurrent outbreaks. These dynamical behaviours emerge from the nonlinear interaction between transmission intensity, renewal of colony-level susceptibility feedback, and the finite capacity of recovery processes.

Throughout this section, the state variables *S*(*t*), *I*(*t*), and *R*(*t*) are interpreted as colony-level subpopulations rather than disease states of individual bees. The model therefore describes aggregate infection burden within a colony, which may arise from continual turnover of individuals, heterogeneous exposure, and repeated environmental contamination. Under this interpretation, recovery does not necessarily represent permanent pathogen clearance at the individual level. Instead, it reflects a temporary reduction in colony infection burden resulting from treatment, mortality of infected individuals, or behavioural or physiological processes that reduce detectable infection prevalence. Reinfection then represents renewed exposure or recolonisation of the parasite within the colony environment. This interpretation is consistent with empirical observations that colony-level Nosema prevalence can decline following intervention but later re-emerge due to environmental persistence and demographic turnover.

**Fig. 5:**
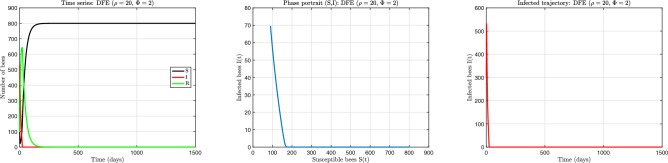
Disease-free equilibrium regime obtained for $(\rho =20,\ \phi =2)$. (**a**) Time evolution of colony composition showing rapid elimination of infection and convergence to the infection-free state. (**b**) Phase portrait in the (*S*, *I*) plane demonstrating monotonic convergence toward the disease-free equilibrium without oscillation. (**c**) Infected population trajectory illustrating complete infection clearance following a short transient adjustment. These dynamics occur when recovery capacity remains sufficiently effective across all infection levels.

**Fig. 6:**
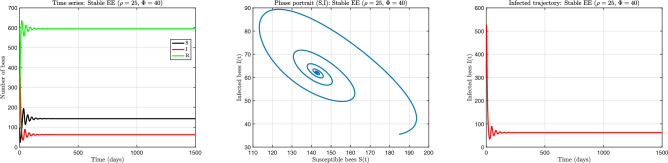
Stable endemic equilibrium regime obtained for $(\rho =25,\ \phi =40)$. (**a**) Time evolution showing damped oscillations that converge to a persistent non-zero infection level. (**b**) Phase portrait displaying spiral convergence toward the endemic equilibrium. (**c**) Infected population trajectory highlighting transient adjustment before stabilisation. This regime represents chronic colony-level infection persistence arising from partial limitation of recovery processes.

**Fig. 7:**
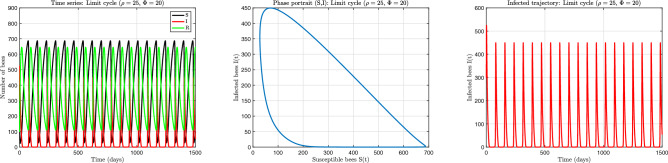
Stable limit cycle regime obtained for $(\rho =25,\ \phi =20)$. (**a**) Time evolution showing sustained periodic fluctuations in colony composition. (**b**) Phase portrait revealing a closed invariant orbit characteristic of persistent oscillatory dynamics. (**c**) Infected population trajectory demonstrating recurrent large-amplitude outbreak cycles. The oscillations are generated by nonlinear feedback between infection growth and saturation of recovery capacity.

The numerical results corresponding to the three principal dynamical behaviours are presented in Figs. 5, 6 and 7. Each figure illustrates temporal evolution of colony composition together with the associated phase-space structure. These simulations demonstrate how nonlinear recovery limitations can produce qualitatively distinct colony outcomes even under constant environmental conditions.

Figure 5 shows the disease-free regime obtained when recovery capacity is sufficiently effective relative to infection pressure, here illustrated by $(\rho =20,\ \phi =2)$. Under these conditions, the infected population declines rapidly and monotonically toward zero. The susceptible class expands to occupy nearly the entire colony, while the recovered class disappears after a transient period because no new infections occur. The phase portrait confirms global convergence toward the infection-free equilibrium. Trajectories move directly toward the boundary $I=0$ without oscillation, indicating that nonlinear feedback is insufficient to destabilise the disease-free state. The absence of transient amplification reflects strong effective recovery relative to transmission. From a biological perspective, this regime corresponds to successful colony-level suppression of infection. Treatment or natural recovery processes operate efficiently enough to remove infection faster than it can propagate or re-establish. Renewal of colony-level susceptibility feedback is present in the model but remains dynamically irrelevant because infection prevalence never reaches levels at which recovery saturation becomes limiting. The colony therefore converges toward a stable infection-free state.

A qualitatively different behaviour emerges when recovery efficiency is reduced relative to infection pressure. Figure 6 illustrates the case $(\rho =25,\ \phi =40)$, where infection persists at a stable non-zero level. The time series shows damped oscillations that gradually decrease in amplitude before stabilising. The phase portrait exhibits a spiral trajectory converging toward a fixed interior point, which confirms local asymptotic stability of the endemic equilibrium. These transient oscillations arise because recovery responds with a delay when infection levels are elevated. As treatment capacity becomes partially saturated, recovery temporarily lags behind infection growth, producing overshoot and subsequent adjustment. However, the feedback is not sufficiently strong to maintain oscillations indefinitely, and the system settles into a steady balance between infection acquisition and removal. Biologically, this regime represents chronic colony-level infection persistence. The parasite is not eliminated, yet it does not generate recurring epidemic waves. Instead, the colony stabilises at a persistent infection burden determined by the balance of transmission, partial recovery, and renewal of colony-level susceptibilty. This behaviour is consistent with long-term endemic prevalence observed in many managed colonies where treatment reduces but does not eradicate infection.

When recovery becomes more strongly constrained relative to infection growth, the system undergoes a qualitative transition from equilibrium behaviour to sustained oscillations. Figure 7 presents the parameter set $(\rho =25,\ \phi =20)$, for which the system exhibits persistent periodic fluctuations. The time series displays repeated large-amplitude infection peaks separated by recovery phases. The phase portrait reveals a closed invariant orbit, demonstrating the existence of a stable limit cycle. These oscillations arise from a self-regulating feedback mechanism generated by recovery saturation. When infection prevalence becomes high, the effective recovery rate declines because treatment capacity cannot increase proportionally. This temporary reduction in recovery allows infection to persist and eventually rebound after partial suppression. As infection falls, recovery capacity becomes more effective again, which reduces prevalence until transmission once more dominates. The repeated alternation of these processes produces sustained endogenous epidemic cycles. Importantly, these oscillations occur in the absence of seasonal forcing or external perturbation. The recurrent outbreaks are generated entirely by internal nonlinear feedback within the colony. This mechanism provides a plausible explanation for repeated infection cycles observed in colonies even under stable environmental conditions.

Taken together, the three dynamical behaviours demonstrate that colony infection dynamics depend critically on the balance between transmission and the effective capacity of recovery processes. When recovery remains sufficiently responsive across all infection levels, infection is eliminated. When recovery is moderately constrained, infection persists at a steady level. When recovery becomes strongly limited at high prevalence, delayed feedback generates recurrent epidemic cycles. These results highlight the important role of nonlinear recovery saturation in shaping colony resilience. The model predicts that increasing infection burden does not necessarily produce a proportional recovery response. Instead, resource or treatment limitations can create delayed regulation that fundamentally alters system stability. As a consequence, small parameter changes can shift the colony between elimination, persistence, and cyclic outbreak regimes.

The ability of a single mechanistic model to generate these qualitatively distinct outcomes supports the hypothesis that renewal of colony-level susceptibility and finite recovery capacity are key drivers of colony-scale disease variability. The time-domain simulations presented here provide direct confirmation of the bifurcation structure analysed in subsequent sections and establish the dynamical foundation for understanding transitions between alternative epidemiological states.

### Sensitivity of dynamical behaviour to model parameters

To quantify the relative influence of epidemiological and demographic processes on the long-term infection burden, we performed a sensitivity analysis of the model output with respect to key parameters. The analysis evaluates how small relative perturbations in each parameter affect a scalar summary of infection dynamics, thereby identifying the mechanisms that most strongly regulate disease persistence within the colony.

Sensitivity was computed using the normalised local sensitivity index

$$\begin{aligned} S_p = \frac{p}{Y}\frac{\partial Y}{\partial p}, \end{aligned}$$

where *Y* denotes the selected infection outcome and *p* is the parameter under consideration. This dimensionless measure represents the proportional change in the infection metric induced by a proportional change in the parameter, allowing direct comparison across parameters with different biological units. Numerical derivatives were approximated using a central finite-difference scheme around the baseline parameter set.

**Fig. 8:**
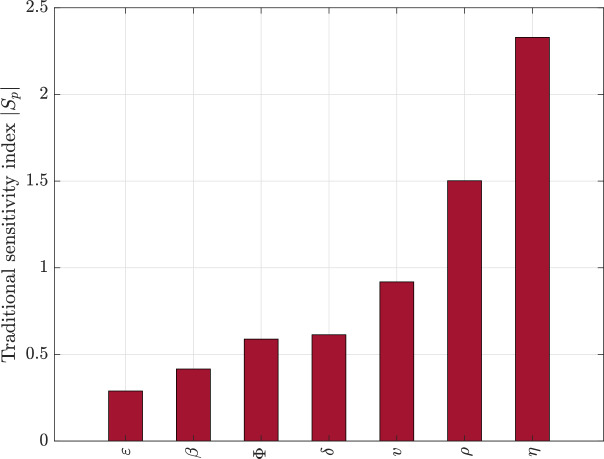
Normalised local sensitivity indices for key model parameters. Bars represent the magnitude of the proportional change in the long-term infection level resulting from a small proportional perturbation in each parameter.

Figure 8 presents the absolute values of the sensitivity indices for the principal model parameters. The results reveal a clear hierarchy in the mechanisms controlling long-term infection behaviour.

The analysis demonstrates that the demographic recruitment rate $\eta$ exerts the strongest influence on infection dynamics. This indicates that the long-term burden of Nosema infection is highly sensitive to the rate at which new susceptible individuals enter the colony. Biologically, this reflects the fundamental role of population turnover in sustaining transmission. Increased recruitment continuously replenishes the susceptible pool, counteracting depletion caused by infection and recovery, and thereby maintaining favourable conditions for parasite persistence. The dominance of this parameter highlights that colony demography is not merely a background process but a primary driver of epidemiological stability.

The treatment or removal intensity $\rho$ emerges as the second most influential parameter. This finding confirms that mechanisms regulating the clearance of infected individuals play a central role in shaping infection outcomes. Because treatment enters the model through a saturating functional response, its effect depends not only on its magnitude but also on the current infection level. Consequently, small changes in treatment capacity can produce disproportionately large shifts in infection persistence, particularly when the system operates near saturation thresholds. This sensitivity underscores the importance of effective management interventions in controlling colony infection levels. Natural mortality *v* also shows a strong regulatory effect. Mortality simultaneously removes susceptible, infected, and recovered individuals, thereby altering both transmission opportunities and population structure. Its relatively high sensitivity indicates that background demographic losses significantly reshape epidemiological feedback loops by modifying host availability and turnover. In ecological terms, mortality acts as a global regulator of population density and therefore indirectly modulates effective contact rates within the colony. The recovery rate $\delta$ and the treatment half-saturation constant $\Phi$ exhibit intermediate influence. Recovery shortens the infectious period and therefore directly reduces transmission potential, but its effect is moderated by renewal of colony-level susceptibility and demographic replenishment processes that reintroduce susceptibility. Similarly, the half-saturation constant governs the efficiency of treatment at different infection levels, shaping how rapidly treatment effects saturate as infection increases. The moderate sensitivity of these parameters suggests that they regulate infection intensity rather than fundamentally determining whether persistence occurs. In contrast, the transmission rate $\beta$ and the renewal of colony-level susceptibility rate $\varepsilon$ display comparatively smaller sensitivity magnitudes under the baseline parameter regime. Although transmission is essential for invasion, once the system operates within a parameter region that supports persistence, marginal changes in transmission intensity produce smaller proportional changes in long-term infection levels than demographic or treatment processes. Renewal of colony-level susceptibility likewise contributes to maintaining susceptibility recycling, but its local influence is weaker than that of mechanisms directly governing host turnover and removal.

Taken together, the sensitivity structure reveals that the long-term behaviour of the system is controlled primarily by demographic renewal and infection removal processes rather than by transmission alone. This finding provides an important mechanistic insight. While transmission initiates outbreaks, the persistence and magnitude of infection are governed more strongly by the balance between host recruitment, mortality, and treatment capacity. In practical terms, this implies that effective management of Nosema infection requires coordinated regulation of colony population dynamics and treatment efficiency, rather than reliance solely on reducing transmission rates. More broadly, the hierarchical organisation of parameter influence confirms that the model dynamics are structurally robust to small perturbations in transmission and renewal of colony-level susceptibility but remain highly responsive to demographic and management-related processes. This sensitivity pattern is consistent with the nonlinear feedback structure of the model, in which sustained infection emerges from the continuous replenishment and removal of hosts interacting with saturating treatment dynamics. The results therefore provide quantitative evidence identifying the key biological mechanisms that regulate the stability and persistence of infection within honeybee colonies.

## Conclusion and implications

This study developed and analysed a nonlinear epidemiological framework to investigate the dynamical consequences of renewal of colony-level susceptibility and treatment saturation in Nosema ceranae infection dynamics. By integrating analytical stability theory, bifurcation analysis, and numerical simulations, the model reveals that infection outcomes are not governed by a single threshold but instead emerge from the interaction of multiple nonlinear feedback mechanisms. These interactions generate a rich spectrum of dynamical regimes, including disease elimination, persistent endemic infection, sustained oscillations, and regions of bistability where long-term outcomes depend sensitively on initial conditions.

A key outcome of the analysis is the identification of oscillatory infection dynamics arising through Hopf bifurcation when treatment capacity becomes effectively constrained relative to transmission and renewal of colony-level susceptibility processes. This demonstrates that recurrent epidemic cycles can arise endogenously from internal system feedback, even in the absence of seasonal forcing or environmental variability. The results therefore provide a mechanistic explanation for recurrent infection patterns frequently observed in managed honeybee colonies. In particular, the combination of renewal of colony-level susceptibility and limited effective control interventions creates a dynamical feedback loop that periodically restores susceptibility and allows infection to resurge. The analysis further shows that the system exhibits bistability within specific parameter regions. Under identical environmental and epidemiological conditions, colonies may converge either to persistent oscillatory infection or to a stable endemic equilibrium depending on the initial infection burden. This path dependence highlights the importance of early infection levels and intervention timing. Once infection crosses a critical threshold, the colony may be driven towards sustained cyclic outbreaks that cannot be reversed by small parameter changes alone. From a dynamical systems perspective, this behaviour reflects the coexistence of multiple attractors separated by basin boundaries, emphasising that disease management is inherently history dependent.

Sensitivity analysis demonstrates that the predicted dynamical regimes are structurally robust across continuous parameter ranges rather than arising from isolated parameter choices. Transmission intensity primarily determines whether infection can invade and persist, renewal of colony-level susceptibility governs the recycling of susceptibility and maintenance of recurrent outbreaks, and recovery regulates infection magnitude while modulating oscillatory amplitude. These findings clarify the functional roles of epidemiological mechanisms and show that long-term infection behaviour is controlled by their nonlinear interaction rather than by any single process acting independently.

From a biological perspective, the results support the interpretation of infection dynamics at the colony scale rather than at the level of individual bees. The recovery and renewal of colony-level susceptibility processes represented in the model capture effective turnover of infection status within the colony population, reflecting replacement of individuals, partial treatment effects, and continual exposure to infectious material. Under this interpretation, the model provides a mechanistic description of how colony-level infection prevalence evolves under sustained environmental pressure and constrained intervention capacity. The emergence of recurrent infection cycles has important implications for disease monitoring and control. Oscillatory dynamics imply that short-term declines in infection prevalence do not necessarily indicate long-term eradication. Instead, infection may re-emerge through endogenous system feedback. This suggests that management strategies based solely on periodic treatment may be insufficient if renewal of colony-level susceptibility pathways remain active or treatment capacity becomes saturated during peak infection periods. The presence of bistability further indicates that early intervention is critical. Colonies with low initial infection burden may remain stable under moderate control effort, whereas colonies that begin with high infection levels may require substantially stronger or sustained intervention to avoid persistent epidemic cycling. This threshold-like behaviour implies that preventive management may be more effective than reactive treatment once infection becomes established. The sensitivity results also emphasise that effective control requires coordinated action across multiple epidemiological mechanisms. Reducing transmission alone may not eliminate recurrent outbreaks if reinfection continues to replenish susceptibility. Increasing recovery through treatment may reduce infection burden without preventing recurrence if treatment capacity is limited relative to parasite load. Long-term stabilisation therefore requires simultaneous reduction of transmission pressure, limitation of renewal of colony-level susceptibility pathways, and maintenance of sufficient treatment effectiveness.

While the present model captures key nonlinear mechanisms governing within-colony infection dynamics, several extensions would further enhance biological realism. Incorporating explicit colony demography, environmental spore reservoirs, or interactions with additional stressors such as nutrition and pesticide exposure would allow investigation of multi-factor drivers of colony health. Coupling the within-colony framework to between-colony transmission processes would also permit analysis of infection spread across apiaries and landscape-scale persistence. Empirical calibration using longitudinal infection prevalence data would provide quantitative validation of parameter ranges and improve predictive capability. Such data-driven integration represents an important direction for future work, particularly for assessing intervention strategies under realistic management scenarios.

In conclusion, the study demonstrates that nonlinear feedback between transmission, recovery, renewal of colony-level susceptibility, and treatment limitation can generate complex and persistent infection dynamics at the colony level. The results provide a coherent dynamical explanation for recurrent outbreaks, regime transitions, and sensitivity to initial infection burden. More broadly, the analysis illustrates how epidemiological systems subject to resource constraints and partial immunity can exhibit multiple long-term outcomes even under constant environmental conditions. By linking mechanistic assumptions to observable dynamical patterns, the model provides a quantitative framework for understanding infection persistence in managed honeybee populations and offers insight into the design of intervention strategies that account for nonlinear system behaviour. These findings contribute to the broader understanding of eco-epidemiological dynamics in host populations where recovery is incomplete and control resources are finite.

## Data Availability

Simulation data and codes will be shared upon reasonable request
